# Designing Potent Anticancer Peptides by Aurein 1.2 Key Residues Mutation and Catenate Cell-Penetrating Peptide

**DOI:** 10.34172/apb.2023.063

**Published:** 2022-12-06

**Authors:** Hamta Salarpour Garnaie, Arman Shahabi, Mohammad Hossein Geranmayeh, Abolfazel Barzegar, Ahmad Yari Khosroushahi

**Affiliations:** ^1^Department of Biophysics, Research Institute for Fundamental Sciences (RIFS), University of Tabriz, Tabriz, Iran.; ^2^Cell Therapy and Regenerative Medicine Comprehensive Center, Kerman University of Medical Sciences, Kerman, Iran.; ^3^Drug Applied Research Center, Tabriz University of Medical Sciences, Tabriz, Iran.; ^4^Department of Medical Nanotechnology, Faculty of Advanced Medical Sciences, Tabriz University of Medical Sciences, Tabriz, Iran.

**Keywords:** Cancer, Anticancer peptides, Aurein 1.2, Cell-penetrating peptide

## Abstract

**Purpose:**

Aurein 1.2 (Aur) peptide is known for possessing anticancer characteristics devoid of conventional therapeutics side effects. For improving Aur peptide anticancer functionality, different anticancer peptides were constructed based on Aur peptide through targeting two separate strategies, including (1) sequence-based mutations and (2) adding a cell-penetrating peptide linker.

**Methods:**

The study was approached by designing three different analogs of Aur, including (a) Aur mutant (Aur_m_), (b) Aur with N-terminal polyarginine linker (R5-Aur), and (c) Aur_m_ with R5 (R5-Aur_m_). Computational molecular dynamics simulations clearly showed higher structural stability of R5-Aur and R5-Aur_m_ compared to Aur, solely. The α-helical properties of R5-Aur and R5-Aur_m_ were protected during 500 ns simulations in water solution while no such structural conservation was seen for Aur *in silico*.

**Results:**

The results of the current study highlight response to one of the main challenges of cancer therapy through selective invasion of Aur to cancer cells without significant involvement of normal cells. This issue was confirmed by different assays, including: MTT assay, flow cytometry, qPCR, and nuclei morphological observations. Furthermore, this study intensifies exploiting *in silico* approaches for adjusting drug delivery. The results of different assessments on designed peptides reveal an anticancer activity pattern rising from Aur toward Aur_m_, and R5- Aur, consecutively.

**Conclusion:**

The designed structure of Aur shows improved anticancer activity through molecular changes which makes it suggestable for anticancer therapies.

## Introduction

 Some antimicrobial peptides have been known for their anticancer activity without showing the usual side effects of conventional therapies such as chemotherapy, radiation therapy, and surgery. One imperative example of these side effects is healthy tissue damage during cancer treatment which allows cancer cells to progress, delocalize, and become resistant against treatments.^1^ However, antimicrobial peptides entail selective toxicity and they are not following common mechanisms of chemical resistance.^2^ Antimicrobial peptides are the essential components of the inherited and acquired immune system which is present in the host defense system against invasions of microorganisms.^2,3^ The antimicrobial activity of this group of peptides is related to their ability to lyse microorganisms through penetrating their membrane.^4^ Important properties of antimicrobial peptides such as selective toxicity, abrupt death induction, wide performance, and lack of becoming microbial resistance have encouraged scientists to exploit these peptides as therapeutics against cancer.^5,6^

 According to the studies performed on the anticancer peptides effects on cancer cells, anticancer peptides with positive amino acids showed a higher tendency for electrostatic interactions with cancer cells which was attributed to the negative charge of cancer cells outer membrane compared to normal cells.^7,8^ Rising alpha-helix conformations with short sequence and hydrophobic structures have been suggested for enhancing anticancer peptides contact regions with cancer cells to destroy them.^9-12^ Aurein 1.2 (Aur) is a 13 amino acid peptide (GLFDIIKKIAESF) with an alpha-helix structure and hydrophilic-hydrophobic segments. Primarily, Aur was isolated from the Australian frog skin. This peptide triggers apoptotic cell death pathways by making pores in the cell membrane or by destroying the mitochondrial membrane after entering the cancer cells. This peptide has a high therapeutic effect on various types of cancers comparing other anticancer peptides. These characteristics of Aur were related to its small size and non-complex structure which highlights it as a suitable biophysical molecule for cancer therapy. Prior assessments by investigating Aur toxicity on red blood cells demonstrated the safety of this molecule for anticancer application without threatening intact cells of the body.^13,14^ These unique properties of the Aur provides it a promising candidate for structural manipulation regarding improving its performance and increasing its selective permeability property to kill cancer cells. The ability of cell-penetrating peptides to enhance drug entrance is a strategy for reducing drug side effects by limiting their exposure. Cell-penetrating peptides are carriers which improve cellular absorption of proteins, peptides, DNA, RNA, and various pharmaceutical agents inside the cell lines.^15^ These peptides have many advantages because of their flexibility, high efficiency, and lower cytotoxicity. In the current study, poly-arginine (R5) was studied as a cell-penetrating peptide. R5 is a short cationic peptide with 5 arginine amino acids which delivers peptides into the cancer cells.^16^

 This study aimed to investigate Aur anticancer activity following enhancing hydrophobicity and positive charges (known as key factors in anticancer activity) through sequence alteration in addition to cell-penetrating peptide sequence to Aur amino acid sequence.

## Materials and Methods

###  Sequence acquisition, alignments, and design 

 Different sequences of aurein, including: Aurein-1.1 (P82386), Aur (P82387), Aurein-2.1 (P69016), Aurein-2.3 (P82390), Aurein-2.4 (P82391), Aurein-2.5 (P69018), Aurein-2.6 (P82393), Aurein-3.1 (P69020), Aurein-3.2 (P69022), Aurein-3.3 (P82396), Citropin-1.1 (P81835), Uperin 3.6 (P82043) were obtained from UniProt database (https://www.uniprot.org/). The amino acid sequences of different aureins were aligned with representatives of Aur using ClustalW implemented in Mega5 with the default parameters of pairwise gap penalties of 10 and 0.1 were assigned for gap opening and gap extension, respectively. The multiple alignment penalties of 3.0 and 1.8 were assigned for gap opening and gap extension, respectively. In some cases, minor adjustments were manually made to achieve the optimized alignments. Mutations were created in the Aur sequence compositions for elevating hydrophobicity and positive charging (Table 1). Aurein and Aurein-related peptide profiles were extracted from the UniProt database and then analyzed. After comparing residues of the mutated peptides some patterns were selected and proposed, including (1) for increasing hydrophobicity by placing isoleucine in position 10 instead of alanine, and (2) for increasing positive charge by placing other lysine peptides at position 11 instead of glutamic acid. In the other analog, the R5 cell-penetrating was added to the first amino acid (glycine). In the last analog, all of these changes such as generating mutations and the addition of R5 were applied.

**Table 1:** Designing mutations and addition of R5 sequence to the Aur peptide

**Mutants**	**Designed mutations**
Aurein 1,2 (Aur)	Gly Leu Phe Asp Ile Ile Lys Lys Ile Ala Glu Ser Phe
Aurein 1,2_mutant (Aur_m_)	Gly Leu Phe Asp Ile Ile Lys Lys Ile Ile Lys Ser Phe
Aurein 1,2_R5 (R5-Aur)	Arg Arg Arg Arg Arg Gly Leu Phe Asp Ile Ile Lys Lys Ile Ala Glu Ser Phe
Aurein 1,2_mutant_R5 (R5-Aur_m_)	Arg Arg Arg Arg Arg Gly Leu Phe Asp Ile Ile Lys Lys Ile Ile Lys Ser Phe

###  Peptides conformational studies by molecular dynamics simulations

 Molecular dynamics (MD) simulations for the main peptide of study (Aur, PDB code 1VM5 determined by solid-state NMR) and its analogs were performed under neutral pH conditions with the GROMACS (Groningen Machine for Chemical Simulations) software version 5.0.4. The amino-terminus of the peptides was acetylated to achieve an uncharged N-terminal before any MD simulations for 500 ns. All simulations are conducted using the GROMOS96 53A6 force field by applying the SPC water model. The systems were considered in water molecules that extend up to 1 nm from any edge of the triclinic box to the solute atoms with periodic boundary conditions in all directions and neutralized by NaCl solution (150 mM). The temperature of the systems is preserved at 300 K by using the Berendsen weak coupling method and pressure is maintained at 1 bar by utilizing Parrinello-Rahman barostat in a constant pressure ensemble. All systems are energy-minimized using the steepest descent method. The minimized systems were equilibrated under NVT (constant volume) and NPT (constant pressure) ensemble conditions, for the time scale of 500 ps.^17^ The visual molecular dynamic (VMD) software version 1.9 and UCSF Chimera was used for observing intracellular peptides.^18^ The behavioral and structural changes of peptides during the simulation period were studied using RMSD, RMSF, and DSSP analysis.

###  Experimental procedure 

 Anticancer activity of the peptides (Aur and designed analogs) was investigated on cancer cell lines, including: 1) SW480 (Colon carcinoma cancer cell line) and 2) HT29 (Human colorectal adenocarcinoma cell line) as well as normal cell lines, including: 3) KDR (Human Kidney Epithelial cell line) and 4) HUVEC (Human Umbilical Vein Endothelial Cell line). All cell lines were purchased from the Pasteur Institute (Tehran, Iran). The cells were grown on the 25 cm^2^ cell culture flasks containing RPMI-1640 medium (Gibco, USA) supplemented with 10% fetal bovine serum (Invitrogen, Carlsbad, CA, USA), sodium pyruvate (1 mM), penicillin G100 U/mL, and streptomycin 100 μg/mL (AppliChem, Darmstadt, Germany) in a humidified atmosphere containing 5% CO2 and 95% air at 37°C.

###  Peptide synthesis 

 Aur and the other peptides were synthesized by Pepmic Co., Ltd., (Suzhou, China) Biotech with 95% of purity or higher.

###  MTT assay

 Cell viability was evaluated by the (2,5-diphenyltetrazolium bromide) MTT assay. Briefly, cell lines were exposed to peptides or 5-fluorouracil (5FU) (13 μL/each well of 96 well plates) as a positive control. Then, IC50 (the required concentration of a drug for 50% growth inhibition in vitro) was achieved by the concentration of 10 µM Aur and other peptides on SW480 cultures. For providing experiments, a seeding density of 1.2 × 10^4^ cells/well of 96-well plate was applied. After 24 hours of treatment, the medium was replaced with 200 μL of fresh growth medium containing MTT solution. After 4 hours of incubation, the medium of each well was carefully removed, and subsequently, 200 μL of dimethyl sulfoxide (DMSO) and 25 μL of Sorenson’s buffer (0.1 M glycine, 0.1 M NaCl, pH 10.5) were used to each well. Then, the plates were incubated for 15 min at room temperature and the absorbance was measured using an ELISA plate reader (Biotek, ELx 800, USA) at 570 nm. The growth inhibitory effects of supernatants were calculated according to the following formula: the growth inhibition ratio (IR%) = [(the absorbance of the control group – the absorbance of the experimental group)/the absorbance of the control group] × 100%.

###  Morphological analysis of the apoptosis

 Morphological changes of the treated cells by Aur and analog peptides were evaluated by DAPI (4.6 diamidino-2-phenylindole) under fluorescent microscopy.^19^ DAPI strongly attaches to the nuclear adenine and thymine bases and facilitates qualitative visualization of intracellular progress of apoptosis.^20^ Briefly, sterile coverslip slides were placed in the six-well plates and 3 ml of growth medium containing 3 × 10^5^ cells were cultured in each well. After 48 hours of incubation, cells were washed with a pre-warmed fresh culture medium. Then, it was fixed by 4% paraformaldehyde inside RPMI for 5 minutes. The fixed cells were washed twice with phosphate buffer saline (PBS) and then permeabilized with PBS containing 0.1% Triton X-100 for 5 minutes at 37°C. The permeabilized cells were stained with 100 μL of DAPI for each coverslip for 3 minutes at room temperature. Finally, the slides were washed with PBS and morphological changes of cell nucleus were detected under fluorescent microscopy (Olympus BX64, Olympus, Japan) equipped with a U-MWU2 fluorescence filter (excitation filter BP 330e385, dichromatic mirror DM 400, and emission filter LP 420).

###  Flow cytometry assay

 For quantitative detection of apoptosis and necrosis in the treated cells, annexin-V and propidium iodide (PI) were applied for staining, respectively. Annexin-V can specifically bind to phosphatidylserine in the presence of calcium which makes necrotic and late apoptotic cells versus early and live cells distinguishable. Also, PI can enter the cells in the final stages of apoptosis and necrosis due to the loss of cell membrane integrity. Then, DNA staining by PI occurs and makes these cells detectable. For providing experiments, cells were seeded in six-well plates (3 × 10^5^ cells/well). After 24 hours of incubation, cultures were treated for 48 h. Then, cultures were prepared for staining using Trypsin-EDTA for detachment. Suspended cells were washed and centrifuged at 900 rpm for 10 minutes at 28°C. All cells were prepared for flow cytometry according to the Annexin V-FITC/PI apoptosis kit instructions. Quadrant settings were fixed with untreated, single-stained controls and copied to dot plots of treated cells. Data analysis was conducted using CELL Quest Pro software (BD Biosciences, San Jose, CA, USA). The experiment was repeated two times with triplicate samples for each experiment. Analyses were performed using 150 000 cells at a rate of 800 cells/s.

###  qPCR analysis

 Gene expression profile among groups assessed by qPCR. Briefly, after 48 h of treatment, cultures were washed using sterile PBS (pH 7.2). Then, total RNA was isolated using RNX-plus solution. The total RNA pellet was dissolved in 50 μL of DEPC treated water. In the following, the quantity and quality of total RNA were assessed by UV spectrophotometry and agarose gel electrophoresis, respectively. An amount of 1μg of isolated RNA was employed for the synthesis of complementary DNA (cDNA) using the Prime Script RT Reagent kit based on the manufacturer’s instructions (TaKaRa, Dalian, Liaoning, China). Then, primers were designed for each particular gene (Table 2). Every experiment mixture (20 μL) was containing 10 μL SYBR Green PCR master mix, 1 μL cDNA (1 μg/μL), 1 μL primer (forward and reverse) and 0.8 μL 6-carboxy-X-rhodamine (ROX as reference dye), and analyzed by StepOnePlus Real-Time PCR System (Applied Biosystems, Foster City, CA, USA). Thermal cycling condition was considered one cycle at 95ºC for 5 minutes followed by 40 cycles at 95ºC for 20 seconds, and annealing temperature of each gene was considered 35 seconds. The data analysis was performed using the Pfaffl method and the threshold cycle (Ct) values were normalized with the expression rate of GAPDH as a housekeeping gene.^21^ All reactions were performed in triplicate and negative controls were included in each experiment.^22^

**Table 2:** Primers sequence

**Gene name**	**Sequence**	**Size (bp)**	**TM ºC**	
			**F**	**R**
Bcl-2	F:5´-GGTGGGGTCATGTGTGTGG-3´	19	60.6	60.1
	R:5´-CGGTTCAGGTACTCAGTCATCC-3´	22		
BAX	F: 5- AACATGGAGCTGCAGAGGAT -3	20	59.8	60
	R: CAGTTGAAGTTGCCGTCAGA -3	20		
Caspase-3	F: 5´- GGTTCATCCAGTCGCTTTGT -3´	20	60.1	60.1
	R: 5´- AATTCTGTTGCCACCTTTCG -3´	20		
Caspase-8	F: 5-ACATGGACTGCTTCATCTGC-3´	20	58.2	58.6
	R: 5´-AAGGGCACTTCAAACCAGTG-3´	20		
Caspase-9	F: 5-GACATGCTGGCTTCGTTTCT -3´	20	60.4	60.3
	R: 5´-CTGGTTTGCGAATCTCTGGT -3´	20		
GAPDH	F: AAGCTCATTTCCTGGTATGACAACG-3’	25	61.6	62.6
	R:5’-TCTTCCTCTTGTGCTCTTGCTGG-3’	23		

###  Statistical analyses

 All data were obtained from at least three independent experiments and are expressed as means ± standard deviations (SD). All of the experimental data were analyzed with the one-way ANOVA analysis of variance, using the statistical package for the social sciences (SPSS Inc., Chicago, IL, USA version 16.0). Statistical significance was set at *P* ≤ 0.05.

## Results and Discussion

###  Root mean square deviation (RMSD) data

**Figure 1:**
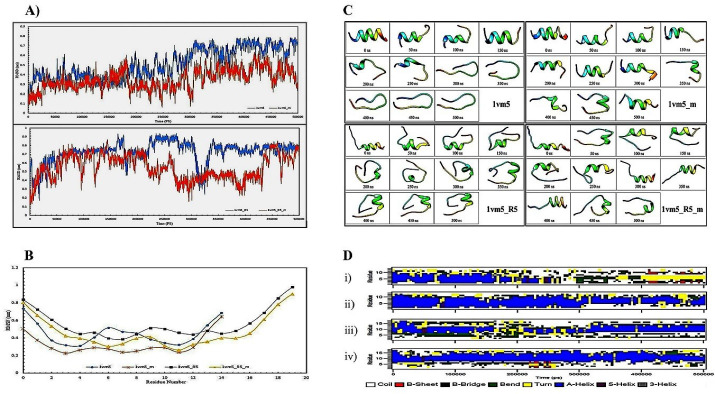
**A)** RMSD data shows peptides alterations for Aur (1vm5) and mutant Aur (1vm5_m) in the upper plot and Aur with R5 sequence (1vm5_R5) and mutant Aur with R5 sequence (1vm5_R5_m) in the lower plot. **B)** RMSF chart for Aur (1vm5), mutant Aur (1vm5_m), Aur with R5 sequence (1vm5_R5), and mutant Aur with R5 sequence (1vm5_R5_m) peptides; **C)** Aur(1vm5), mutant Aur (1vm5_m), Aur with R5 sequence (1vm5_R5), and mutant Aur with R5 sequence (1vm5_R5_m) structure from the beginning to the end of the simulation in every 50 nanoseconds time intervals; **D)** Second structure profiles of the Aur (i), mutant Aur (ii), Aur with R5 sequence (iii), and mutant Aur with R5 sequence (iv) in the simulation period; Aurein 1.2 (Aur), root mean square deviation (RMSD), root mean square fluctuation (RMSF)

 One of the proper indicators of examining overall structural changes during simulation is an RMSD parameter which indicates particle position deviation relative to a reference at each time step. Higher RMSD for one or more atoms during the simulation means higher conformational changes in the molecule structure.^23^ In the current study, the overall changes in the peptide backbone were examined with its prior state. The RMSD fluctuations for Aur_m_ (1vm5­_m) peptide is less than Aur (1vm5) which is related to amino acids replacement in the 10th (Ala → Ile) and 11th (Glu → Lys) leading to higher stability in the molecule structure (Figure 1A). The range of RMSD variations for Aur is 0.2-0.8 nm and for Aur_m_ is 0.1-0.5 nm. Because of the high RMSD fluctuations of R5-Aur (1vm5_R5) and R5-Aur_m_ (1vm5_R5_m) peptides, they reveal unstable structures. It is caused by the presence of the five amino acid sequences called R5. However, the rate of these fluctuations in the R5-Aur_m_ is less than the R5-Aur peptide.

###  Root mean square fluctuations (RMSF) data 

 RMSF is a measure of atoms or amino acids flexibility during the simulation process. The low amino acid fluctuations indicate structural stability and the binding tendency of the amino acid to its ligand.^24^ Our result showed that the highest level of RMSF in all four peptides is related to the Phe amino acid at the ending region (Figure 1B) and the lowest level of RMSF is different for each of the peptides. So, the lowest amount of RMSF for Aur, Aur_m_, R5-Aur, and R5-Aur_m_ are Asp4, Phe3, Leu7, and Ile11 amino acids, respectively. Aur_m_ has fewer fluctuations than Aur. Also, R5-Aur_m_ has fewer fluctuations than R5-Aur. Generally, among these four peptides, the amino acid fluctuations of Aur_m_ and R5-Aur_m_ peptides are lower than the rest of the two other peptides. RMSD data analysis during simulation reveals that mutations in the 10th and 11th amino acid regions of Aur analogs causes decline in structural changes and amino acids flexibility as well as higher stability.

###  Define the secondary structure of protein (DSSP) data 

 This parameter is used to determine temporal variations of the protein secondary structures. DSSP algorithm is defined by the calculation of the presence/absence probability of the existing protein structures percentage during the simulation procedure. So, total A-Helix + B-Sheet + B-Bridge + Turn is identified as structures by the DSSP algorithm.^25^ Our data obtained from the DSSP analysis demonstrates (Figure 1D) Aur (i) almost retains its alpha-helix structure until 200 nanoseconds, and then the structure of the peptide clutters, bends, and turns. Then, B-Sheet, and B-Bridge structures are slightly created. In the Aur_m_ (ii), alpha-helix is dominated in the second structure till the end of 500 ns. While B-Sheet and B-Bridge structures could not be detected. The R5-Aur_m_ (iv) has maintained its alpha-helical structure during simulation more than R5-Aur (iii). In general, peptides ii, iii, and iv show a more stable structure than Aur (i).

 Peptides structure from the beginning to the end of the simulation in every 50 nanoseconds time intervals depicted in Figure 1C. Unlike Aur, the alpha-helicity structure of the Aur_m_ has been retained until the end of the simulation. Also, the R5-Aur and R5-Aur_m_ showed stable structures until the end of the simulation. Since the structural stability of peptides directly affects their bioavailability, it is expectable for peptides to acquire anticancer effects by rising patterns from Aur_m_ to R5-Aur and the R5-Aur_m _peptide. For confirming these results and investigating their accuracy, experimental procedures were provided which were shown in the next sections.

###  Cell death detection by MTT colorimetric assay

**Figure 2:**
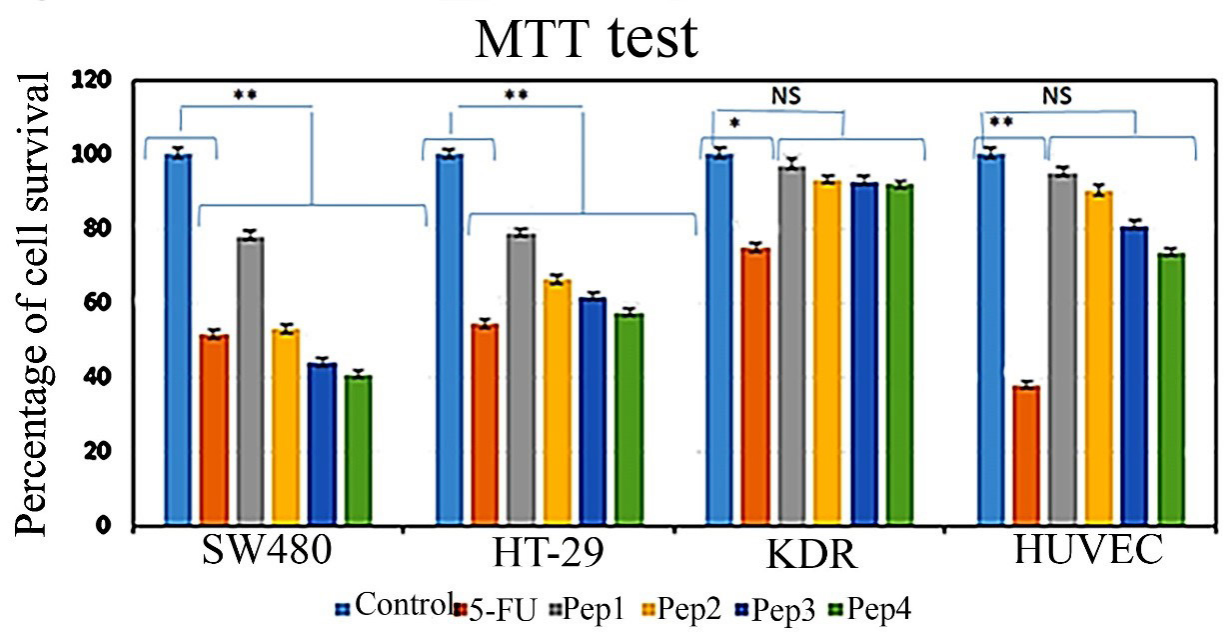
Effect of peptides treatment on cancer cell lines (SW480 and HT29) and normal cell lines (KDR and HUVEC) cultures cytotoxicity comparing 5FU (positive control). Aurein 1.2 peptide (Pep1), mutant Aurein 1.2 (Pep2), Aurein 1.2 peptide with R5 (Pep3), and mutant Aurein 1.2 with R5 (Pep4), non-significant (NS), * *P* ≤ 0.05, ** *P* ≤ 0.01

 MTT assay was analyzed after 48 h of cultured cells treatment by peptides. The results show a slight toxic effect of Aur (Pep1) on SW480 cancer cell cultures (77.87% viability) comparing other peptides (Figure 2); Aur_m_ (Pep2) (51.63% viability rate) induced toxicity was similar to 5FU (52.92% viability); R5-Aur (Pep3) and R5-Aur_m_ (Pep4) were seen to be more toxic than 5FU (43.89% and 40.62% viability, respectively) in SW480 cells culture. Similarly, HT29 cancer cell cultures showed reduced cell population after Aur treatment (78.81% viability) while the cell death rate was higher after treatment by Aur_m_ (66.10% viability) and R5-Aur (61.56% viability). R5-Aur_m_ showed a cytotoxic effect (54.22% viability rate) similar to 5FU (57.34% viability). In the SW480 cultures, Aur_m_ group, and HT29 cultures R5-Aur_m_ group similar cytotoxic effect of 5FU was detected. Furthermore, the R5-Aur and R5-Aur_m_ groups of SW480 cultures presented more cytotoxic effects than the 5FU group.

 In the HUVECs culture, the cytotoxic effect of 5FU was very high (37.85% viability rate), while the cytotoxic effects of the peptides were insignificant. The viability rate following treatment with Aur, Aur_m_, R5-Aur, and R5-Aur_m_ peptides was 95.08%, 89.97%, 80.77%, and 73.62%, respectively. Likewise, for the KDR cell cultures, the cytotoxic effects of peptides were negligible. The percentage of KDR cells population viability as a result of treatment with peptides Aur, Aur_m_, R5-Aur, and R5-Aur_m_ peptides was 96.95%, 93.10%, 92.58%, and 91.77%, respectively. Therefore, the MTT assay result is highly corresponding to the bioinformatics data prediction which indicated designed peptides have higher anticancer efficacy than Aur alone.

###  Qualitative apoptosis detection by DAPI

**Figure 3:**
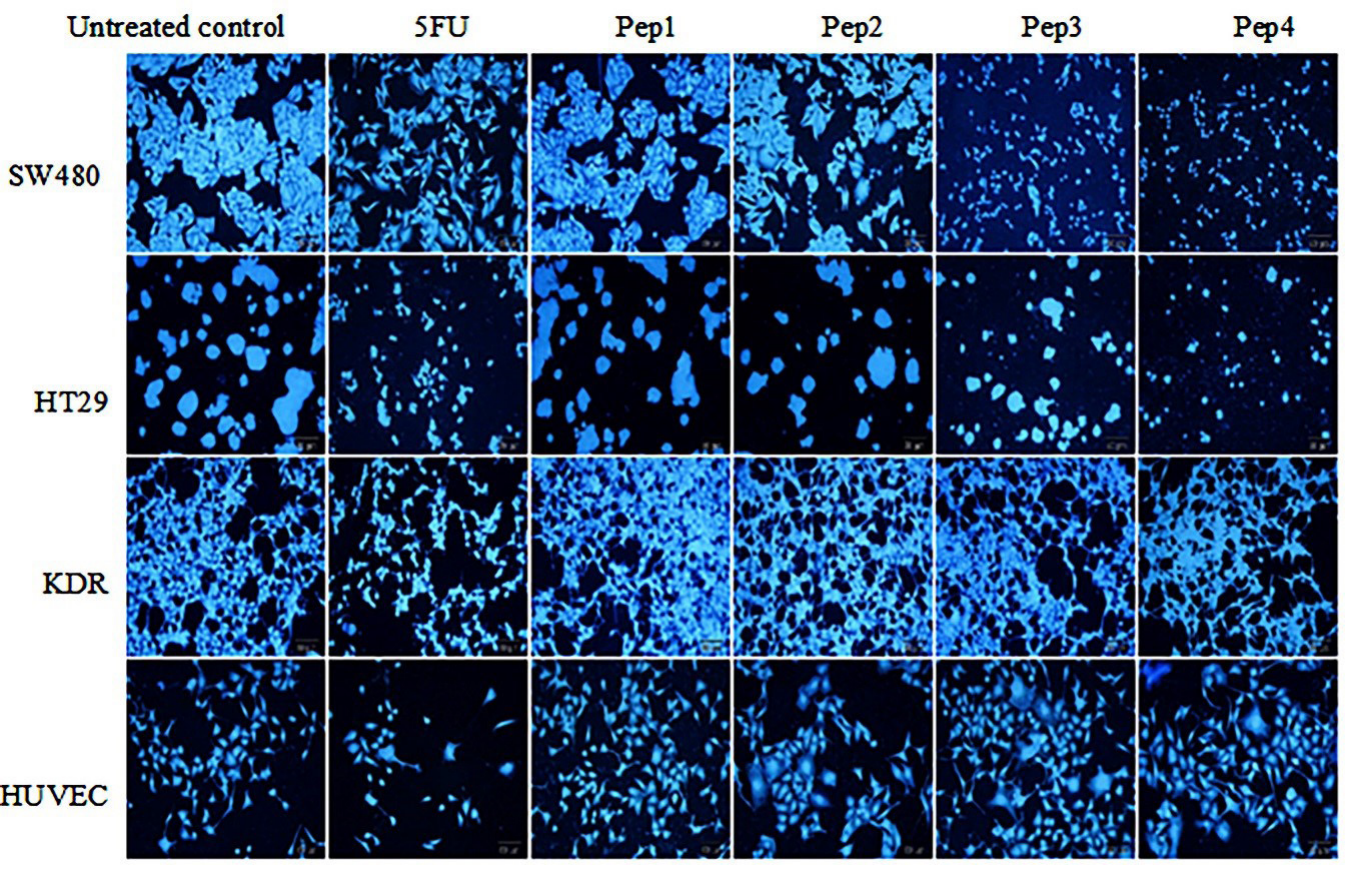
DAPI staining of cultures treated by peptides after 48 hours under fluorescence microscopy. SW480 and HT29 cultures (Cancer cell lines); KDR and HUVEC cultures (Normal cell lines). Pep1 (Aurein 1.2), Pep2 (mutant Aurein 1.2), Pep3 (Aurein 1.2 peptide with R5), and Pep4 (mutant Aurein 1.2 with R5); magnification = 200x

 Exploring DAPI staining under fluorescent microscopy discloses a slight inhibitory effect of Aur on SW480 cell line compared to the control group (Figure 3). While Aur_m_ represents a close appearance to 5FU treated group. Similarly, R5-Aur and R5-Aur_m_ groups showed higher inhibitory effects on cell proliferation than 5FU treated groups. Also, In the HT29 cell culture, R5-Aur and R5-Aur_m_ treatment groups showed cytotoxic effects similar to 5FU treated groups. Conversely, Aur and Aur_m_ have not remarkably affected cell growth which is observable with the presence of low apoptotic cells. The results of KDR cultures confirm MTT assay findings, as peptides do not induce significant cytotoxic effects after treatment, however, many apoptotic cells appeared after treatment with 5FU. While a high apoptotic effect of 5FU group is visible on the nucleus and the cell membrane of the HUVEC culture, the cytotoxic effects of the peptides are lower in HUVEC cultures. Also, the results indicate HUVEC is more sensitive compared to KDR cultures.

###  Detecting apoptosis/necrosis by flow cytometry

**Figure 4:**
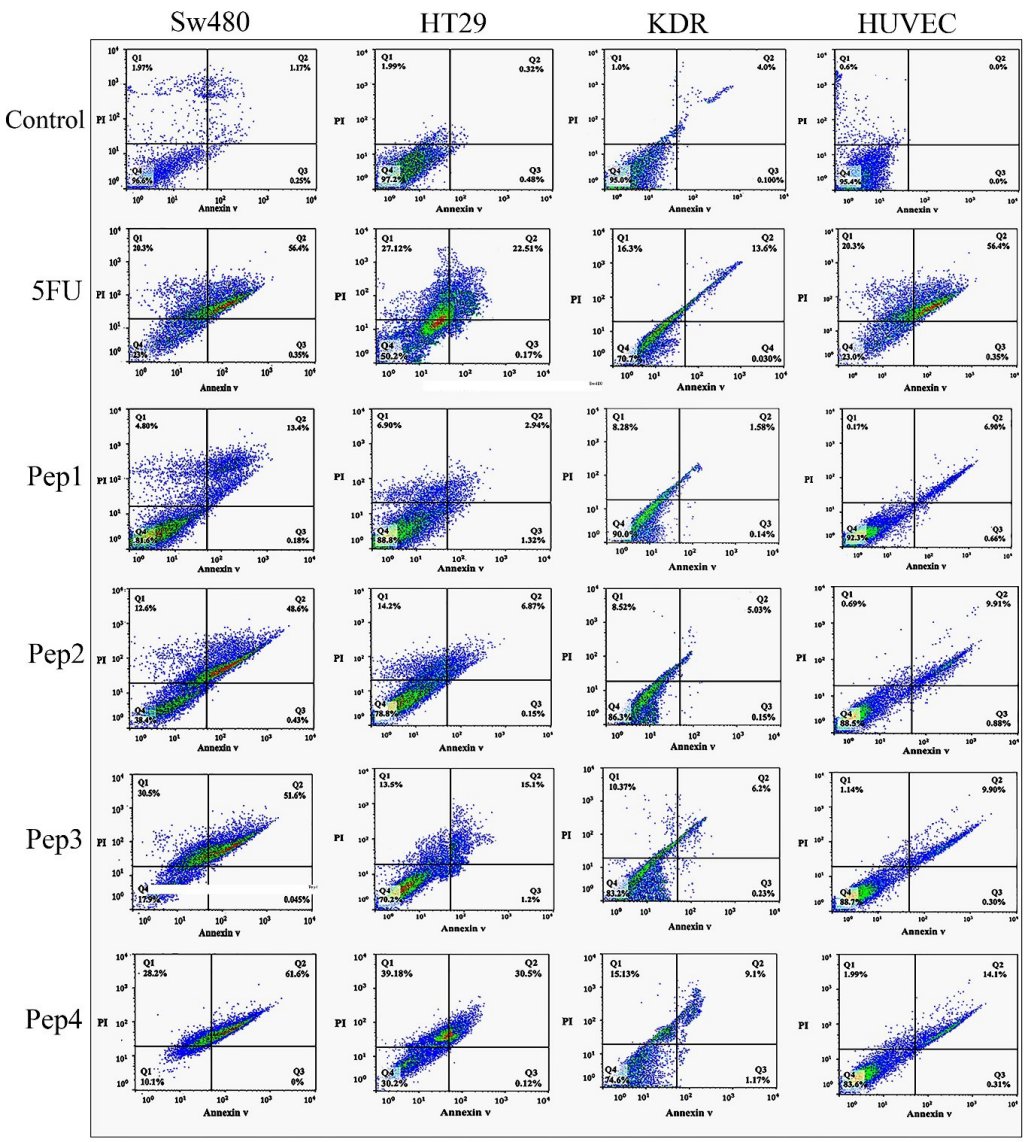
Flow cytometry analysis of cellular staining by Annexin V-FITC and PI after 48 h of treatment with peptides. SW480 and HT29 cultures (Cancer cell lines); KDR and HUVEC cultures (Normal cell lines). Pep1 (Aurein 1.2), Pep2 (mutant Aurein 1.2), Pep3 (Aurein 1.2 peptide with R5), and Pep4 (mutant Aurein 1.2 with R5), Propidium iodide (PI)

 Flow cytometry analysis for detecting annexin V-FITC and PI cellular staining was provided for evaluating live, early/late apoptotic, and necrotic cells population. The results showed severe cytotoxicity in the SW480 cultures treated with Aur_m_, R5-Aur, and R5-Aur_m_ peptides (Figure 4). The toxic effects of R5-Aur, and R5-Aur_m_ were higher than 5FU in the SW480 cultures. The toxicity of Aur_m_ in SW480 cells was similar to 5FU, however, Aur_m_ showed a lower necrosis rate compared to the 5FU treatment group. HT29 cultures showed cytotoxicity in the R5-Aur_m_ peptide group. Finally, the results for KDR and HUVEC cultures were similar to MTT and DAPI observations which indicated peptides cytotoxicity for normal cell lines was slight with a lower necrosis rate than 5FU.

###  Apoptotic genes expression profile 

**Figure 5:**
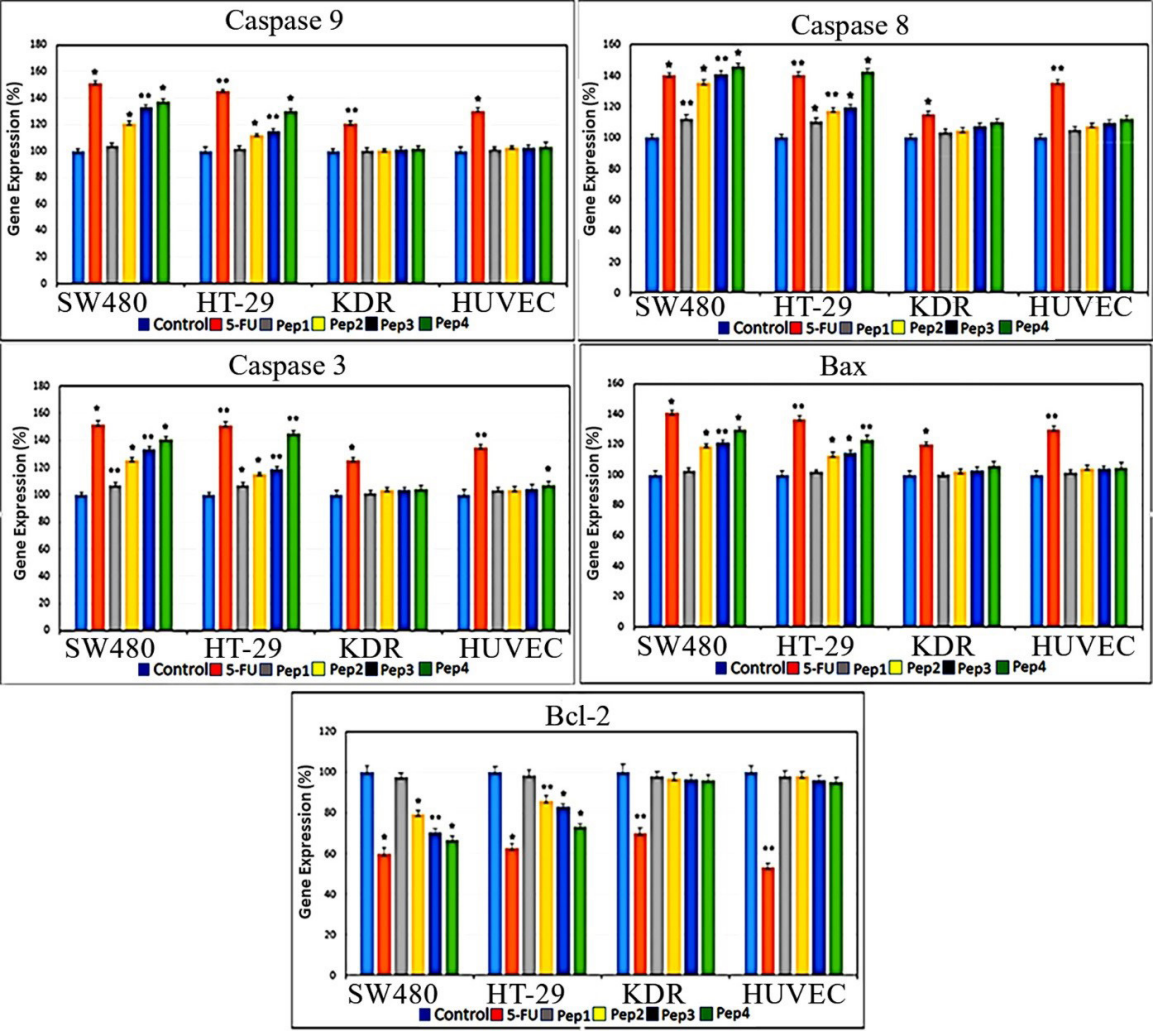
qPCR profile of caspase 3, caspase 8, caspase 9, Bax, and Bcl2 genes after 48 hours of peptides treatment. SW480 and HT29 cultures (Cancer cell lines); KDR and HUVEC cultures (Normal cell lines). Pep1 (Aurein 1.2), Pep2 (mutant Aurein 1.2), Pep3 (Aurein 1.2 peptide with R5), and Pep4 (mutant Aurein 1.2 with R5); * *P* ≤ 0.05 and ** *P* ≤ 0.01

 The real-time gene expression profile for caspase 3 and caspase 9 indicates a rising effect in both of the HT29 and SW480 cancer cell lines treated by Aur_m_, R5-Aur, and R5-Aur_m_ peptides (Figure 5). Caspase 9 gene expression after 5FU treatment was elevated in cancer cell lines. In the normal cell lines, 5FU treated KDR cultures showed caspase 3 gene overexpression and in the HUVEC cultures caspase 3 upregulation occurred in 5FU and R5-Aur_m_ treated groups. In all cell line groups, caspase 8 expression was significantly increased in all treatment groups compared to control. Investigating pro-apoptotic Bax gene expression showed a significant rise in the HT29 and SW480 cultures treated by Aur_m_, R5-Aur, and R5-Aur_m_. Conversely, KDR and HUVEC cultures treated by peptides did not show Bax gene overexpression. Correspondingly, the anti-apoptotic Bcl2 gene expression profile showed a decline after 5FU, Aur_m_, R5-Aur, and R5-Aur_m_ treatment in the HT29 and SW480 cultures. In contrast, in normal cells only in the 5FU treated group, Bcl2 gene expression showed a significant decline.

 Membranolytic anticancer peptides are known for their efficacy in preventing resistant cancer cells generation. Aur, a 13 amino acid peptide, presented its anticancer activity in many types of cancer cells.^21,22,26,27^ The anticancer activity of Aur has been attributed to the cancer cells anionic lipid layer adhesive property. This negative charging occurs through phosphatidylserine accumulation in the outer layer of cancer cells membrane.^24^ The results of the current study highlight the response to one of the main challenges of cancer therapy through Aur selective invasion to cancer cells without significant involvement of normal cells. This issue was confirmed by different assays, including colorimetry (MTT assay), cell sorting (Flow cytometry), quantitative gene expression (qPCR), and morphological observations (DAPI nuclear staining). Furthermore, this study intensifies applying *in silico* approaches for adjusting drug delivery. Comparing different assessments among designed peptides, indicates a rising anticancer pattern for Aur, Aur_m_, R5-Aur, and R5-Aur_m_, consecutively. Also, normal cell lines mild toxicity along with augmenting anticancer outline for these peptides was observable. Despite slight toxicity for normal cells, the results for peptides were promising compared to 5FU treated groups. This issue was more remarkable for the HUVEC cell line viable population (non-apoptotic/non-necrotic) treated by peptides compared to the 5FU group (Figure 4). The peptides discriminated targeting between cancer cell lines can be attributed to the differences in the cell membrane surface, structure, and fluidity. Previously, Aur anticancer activity was demonstrated on T98G human Caucasian glioblastoma and breast cancer cell lines (MCF-7 and Mx-1).^21,24^ Generally, a significant increase in the expression of caspases, after 48 hours of peptides treatment, indicated the activation of both of the internal and external apoptotic pathways. Our results confirm previously described mechanisms for Aur anticancer activity, including necrotic activity via cell membrane lysis and apoptotic activity through disrupting mitochondrial membrane.^28^ The necrotic activity was seen in the PI-positive cellular population in Figure 4 which confirms the reported membranolytic activity of the peptides. Correspondingly, targeting mitochondrial membrane damage is observable in gene expression profile (Figure 5, Bax ↑ and Bcl-2 ↓) by pro-apoptotic consequences (caspase-9 ↑ and caspase-3 ↑). Some studies suggested glycosylation modifications for targeting cancer cells by Aur. As glycosylation rearrangement occurs on the outer layer of cancer cells, N- or O-glycosylation is reported for amplified Aur binding to breast cancer cells.^21^

## Conclusion

 Advances of simulation empowers the utilization of natural molecules inherent capacities for cancer therapy purposes.^29^ Generating mutations and changes such as increasing hydrophobicity by replacing Isoleucine amino acid, enhancing positive charge by replacing lysine amino acid, and adding cell-penetrating peptide sequence (R5) in the Aur anticancer peptide optimize Aur molecule anticancer activity. These effects lead to cancer cells structural stability changes and enhancing membrane permeability. The designed analogs in some cases displayed toxic capacity to eliminate cancer cells more than 5FU anticancer drug. More importantly, these peptides demonstrated low cytotoxic effects on normal cell lines. Therefore, by studying the factors involved in the biological activity of anticancer peptides and reducing their limitations, new anticancer drugs could be designed with lower side effects of conventional therapies on normal cells and increased anticancer activity.

## Acknowledgments

 The financial support of the Iran National Science Foundation (INSF) (grant No.: 95816911) and Tabriz University of Medical Sciences is gratefully acknowledged. This project is a part of M.Sc. thesis conducted at the Drug Applied Research Center, Tabriz University of Medical Sciences, Tabriz, Iran.

## Competing Interests

 There was no conflict of interest to be declared.

## Ethical Approval

 Not applicable.
